# Activating schoolyards: study design of a quasi-experimental schoolyard intervention study

**DOI:** 10.1186/s12889-015-1828-9

**Published:** 2015-05-31

**Authors:** Henriette Bondo Andersen, Charlotte Skau Pawlowski, Hanne Bebendorf Scheller, Jens Troelsen, Mette Toftager, Jasper Schipperijn

**Affiliations:** Research unit for Active Living, Department of Sports Science and Clinical Biomechanics, University of Southern Denmark, Campusvej 55, 5230 Odense M, Denmark; Centre for Intervention Research in Health Promotion and Disease Prevention, National Institute of Public Health, University of Southern Denmark, Øster Farimagsgade 5a, 1353 Copenhagen K, Denmark; Danish Cancer Society, Department of Prevention and Information, Strandboulevarden 49, 2100, Copenhagen Ø, Denmark; National Institute of Public Health, University of Southern Denmark, Øster Farimagsgade 5a, 1353 Copenhagen K, Denmark

**Keywords:** Study design, Participatory intervention development, Mixed method, Schoolyards, Physical activity, GPS, Accelerometer, Observations, Go-along interview, Process

## Abstract

**Background:**

The aim of the Activating Schoolyards Study is to develop, implement, document and assess a comprehensive schoolyard intervention to promote physical activity (PA) during school recess for primary school children (grade 4-8). The intervention is designed to implement organizational and structural changes in the physical environment.

**Method:**

The study builds on a quasi-experimental study design using a mixed method approach including: 1) an exploratory study aimed at providing input for the developing process; 2) an evaluation of the effect of the interventions using a combination of accelerometer, GPS and GIS; 3) a process evaluation facilitating the intervention development process and identifying barriers and facilitators in the implementation process; 4) a post-intervention end-user evaluation aimed at exploring who uses the schoolyards and how the schoolyards are used. The seven project schools (cases) were selected by means of an open competition and the interventions were developed using a participatory bottom-up approach.

**Discussion:**

The participatory approach and case selection strategy make the study design novel. The use of a mixed methods design including qualitative as well as quantitative methods can be seen as a strength, as the different types of data complement each other and results of one part of the study informed the following parts. A unique aspect of our study is the use of accelerometers in combination with GPS and GIS in the effect evaluation to objectively determine where and how active the students are in the schoolyard, before and after the intervention. This provides a type of data that, to our knowledge, has not been used before in schoolyard interventions. Exploring the change in behavior in relation to specific intervention elements in the schoolyard will lead to recommendations for schools undergoing schoolyard renovations at some point in the future.

## Background

Physical activity (PA) in childhood is associated with a multitude of positive short- and long-term health consequences due to its stimulating influence on physical conditions, cognitive performance and mental well-being [1–5]. In spite of the growing awareness of these benefits, a large number of school children do not reach the recommended minimum level of 60 min of moderate-to-vigorous physical activity (MVPA) per day in Denmark and other western countries [6, 7]. In addition, an increase in sedentary time is worrying due to the associations with obesity and metabolic risks, independent of the amount of PA [8]. Since both the PA and sedentary behavior pattern in childhood are likely to track into adulthood, the importance of promoting PA and reducing sedentary behavior in childhood is evident [9–12].

Schools, in particular during recess, are key settings to promote PA because of their potential to reach and influence a large number of students with different backgrounds and PA patterns [13, 14]. Recess PA can contribute with up to 40 % of children’s recommended daily PA [13], and especially for the least active children recess PA has shown to be important [15, 16]. Furthermore, recess PA has been shown to improve cognitive performance, academic achievement, classroom behavior, attention and concentration [12].

Previous recess-based PA interventions have reported mixed results [17–21] and the level of evidence does not seem sufficient to draw conclusions on the intervention effects. Some short-term follow up interventions have shown promising results in increasing PA, *e.g.* adding equipment, playground markings, teachers involvement, and planned activities [18]. However, these results may have captured a novelty effect. More work is needed from different countries in this area, particularly as the structure of recess and implementation of interventions varies within and between countries. Overall there is a growing demand for publishing intervention strategies with an elaborate description of intervention components [19, 22] and long-term follow-up studies are warranted [18, 19].

We developed the Activating Schoolyards Study as a quasi-experimental intervention study with a long-term follow-up. The study is designed to develop, implement, document and assess a comprehensive schoolyard intervention to promote PA in recess for school children (grade 4-8), with a focus on the least active students. The intervention was developed using a participatory approach together with the involved schools and was tailored to the needs of particular schools.

Based on findings from previous intervention studies [23–25] we hypothesized that a high degree of user-involvement, tailored inventive interventions and sufficient funding would lead to increased PA among students. However, exploring and evaluating the effect of the highly tailored interventions requires a special study design. This paper will present the study design, case selection, intervention development, and measurements to be used in the Activating Schoolyards Study.

## Method

### Setting

#### Partnership

A partnership consisting of The Danish Cancer Society, The Danish Foundation for Culture and Sport Facilities, and the Danish foundation Realdania had the vision to increase PA in primary schools in Denmark by redesigning and renovating schoolyards. Together they launched the Activating Schoolyards Campaign. The campaign had a budget of approximately 8 million USD, including 2 million USD of local co-funding. The Danish Cancer Society funded the development of study and the scientific assessment. The Partnership appointed a campaign secretariat that was responsible for all practicalities involved in the recruitment process.

#### Primary schools in Denmark

In Denmark school is mandatory for children between the age of 6 and 16 years. Public schools are free of charge and students do not wear school uniforms. Schools are typically divided into junior (0-3 grade, 6-9 years old), middle (4-6 grade, 10-12 years old) and senior (7-10 grade, 13-16 years old) tiers [26]. Each class has a maximum of 28 gender-mixed students. Students participating in this study attend school for 33 (grade 4-6) and 35 (grade 7-9) hours per week. Approximately 60 min are allocated to recess per day, being distributed over two to four recess periods [26]. In general, the lunch break is the longest recess, lasting 25-30 min. Recess is typically characterized by free play without any organized curriculum. Teachers on yard duty are supervising the students handling conflicts and accidents. Some schools organize ‘Play patrols’ with middle block students organizing games to activate junior students. The junior students must often stay outdoors during recess. There is wide variation in whether schools have an outdoor recess policy for middle-and senior tier students. Seniors are allowed to leave school during recess at most schools.

### Study design

**Figure 1:**
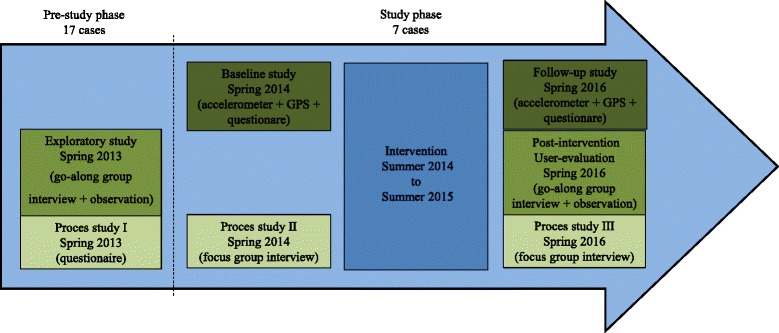
Illustration of study design, timeline and methods

The design is based on a quasi-experimental long-term follow-up study of students attending selected primary schools (grade 4-8) in Denmark. To be able to accommodate both an exploratory and an evaluating part of the study, a range of qualitative and quantitative methods were employed to facilitate exploration and evaluation. The Activating Schoolyards Study is divided into four main parts: 1) exploratory study; 2) effect evaluation; 3) process evaluation; 4) post-intervention user-evaluation. The studies were divided into two different phases: 1) the project development pre-study phase and; 2) the study phase. The aim of the studies conducted in the pre-study phase was to provide input and create inspiration for the interventions. The aim of the study phase was to evaluate the Activating Schoolyards Study in terms of effect, process, and user-perspective. The study design with its different sub-studies and phases is illustrated in Fig. 1.

### Case selection

**Figure 2:**
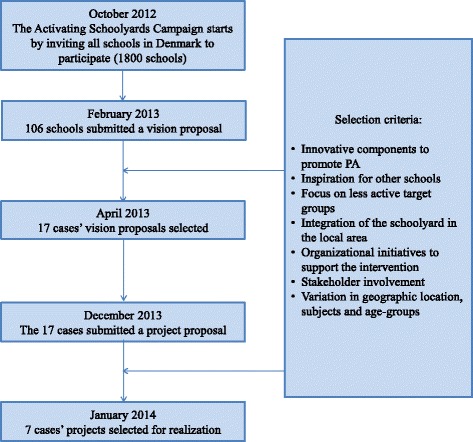
Flow diagram of case selection

The project schools (cases) were selected by means of an open competition in order to stimulate local engagement and participation in the development of the interventions [23]. In October 2012, all primary schools in Denmark (approximately 1800) were invited to submit a vision proposal for improvement of their schoolyard. Out of the 106 submitted proposals, 17 cases were selected for further development in April 2013 by an evaluation panel appointed by the Partnership. Each of these 17 cases received approximately USD 17,000 to further develop their vision in self-constituted case teams comprised of external consultants chosen by the schools (architects, landscape architects, designers) and stakeholders (students, teachers, parents, neighbors, and local organizations). The 17 project proposals were submitted in December 2013, and in February 2014 the evaluation panel selected seven cases for realization. The case selection process is presented in Fig. 2.

The evaluation panel selected both the vision proposal and the final project description to favor the following selection criteria: innovative solutions promoting PA, inspiration to other schools, focus on less active target groups, integration of the schoolyard in the surrounding local area, organizational initiatives to support the intervention, student and stakeholder involvement, and diversity of locations and target groups. The selected projects had to document that they could provide at least 50 % of the budgeted cost of the project. The total budget for each of the projects ranged from 120,000 to 900,000 USD. The seven cases represent a wide range of schools. As shown in Table 1, the seven cases differed considerably in geographical area, school type (urban or rural), number of students enrolled (middle and senior tiers), socioeconomic status (based on parental income), share of students with a non-Danish ethnicity, square meters of schoolyard per child, number of play facilities, recess duration, number of playground duty teachers, recess rules, and organized play activities during recess.

**Table 1:** Case characteristics regarding the study target group; middle and senior block students

Case	Geographical area	School type	No. on roll	Parents income range*	Share with a non-Danish ethnicity (%)	Size of school-yard (m^2^)	Size of school-yard per child (m^2^)	No. of play facilities	Recess periods + duration (min.)	No. of duty teachers	Outdoor recess policy	Mobile phone during recess	Recess PA initiatives
1	Region Zealand	Urban	457	-	20	6888	15	15	20	3	No	Yes	Play patrol***
									30				
									5				
2**	Capital Region	Urban	174	< Average income	25	3902	22	15	20	4	No	Yes	Play patrol
									30				
									5				
	Capital Region	Urban	424	< Average income	14	6767	16	15	15	4	Yes (middle block during summer)	Yes	
									40				
									10				
3	Region North	Rural	418	> Average income	0	59333	142	16	20	4	Yes (middle block)	Yes	
									30				
4	Region North	Urban	406	> Average income	14	33415	82	20	30	5	Yes (middle block)	Yes	Play patrol Sports hall use
									30				
									5				
									5				
5	Central Denmark	Rural	186	< Average income	1	13311	72	11	30	2	No	Yes	Teacher initiated activities
									25				
									10				
6	Southern Denmark	Rural	59	> Average income	3	26314	120	27	10	4	Yes	No	Play patrol Sports hall use
									40				
									25				
7	Region Zealand	Rural	45	> Average income	0	6747	73	13	15	2	Yes	Yes	Play patrol
									40				
									5				

*Published data from Statistics Denmark. One school is not included why it has been merged after the calculation

**Case = the project school. Case 2 includes two schools

***Play patrol = middle block students educated to activate junior students with structured games (voluntary participation)

### Development of interventions

The interventions were developed using a participatory bottom-up approach inspired by Community-Based Participatory Research ideas [27]. Building on existing capacities in the ‘case’ community, the interventions (*e.g.* target groups, areas and components) submitted in the project proposals reflected local challenges and needs. The interventions contain both physical and organizational changes. During the intervention development process, all case teams had access to a campaign website that provided various materials for inspiration including a large number of short thematic case descriptions of other schoolyard renovation projects, as well as short videos with interviews with students, school principals and researchers. The case teams were also obliged to attend two workshops. In May 2013, a start-up workshop was conducted for the 17 case teams aiming to provide inspiration, stimulate innovation and share knowledge from previous schoolyard interventions. Moreover, findings from the exploratory study on the students’ perceived barriers for recess PA were presented at this workshop to inspire the development of the organizational changes. A second workshop was organized for the seven case teams in February 2014 focusing on qualifying and anchoring the projects, and providing inspiration for organizational initiatives. Furthermore, the process evaluation was designed to help the case teams think through the decisions made during the intervention development.

It was left up to each case team to decide if and how the provided information and feedback could be incorporated. The whole process led to highly tailored interventions with considerable variation in intervention components between the seven cases. In some of the cases the interventions took place in the existing schoolyard whereas other cases expanded their outdoor area by including adjacent spaces (*e.g.* forests and streets). Even though the design and dimension of the intervention components varied widely, some features were present in several cases, *e.g.* the introduction of climbing walls, balance-bars, amphitheater-stages, skating areas, trampolines, and outdoor lunch eating areas. There were also similarities in the planned organizational changes, *e.g.* implementation of a movement policy and changes in recess duration. An overview of the intervention elements per case can be found in Table 2. All interventions will be implemented between summer 2014 and summer 2015.

**Table 2:** Planned intervention components

Case target group	Physical interventions	Organizational interventions
1	Rebuilding a flat asphalt covered schoolyard adding five movement areas: 1. The Hill is 3.5 m tall covered with a climbing wall. Below the hill is a dancing area 2. The Music area is an in-ground-amphitheater beside with three trampolines. 3. The Moat area is an outdoor classroom surrounded by an 80 m^2^ rein-bed 4. The Playground kitchen is an outdoor canteen. 5. The Play-box is a multi-court	• Movement policy
Grade 7-9		• New recess rules
		• Activities in the lessons
		• After school activities
2	Closing a suburban street between two schools and transform it into areas for movement and places to hangout. The street will frame five areas for activity connected by a bicycle lane/walking path: 1. An angled climbing wall 2. An in-ground mini-court 3. Stumps of concrete 4. A four squared rubber-surfaced area shaped as a tribune with a climbing area. 5. Four sloping asphalt surfaces with soccer-golf on the sides.	• Movement policy
Grade 7-9		
3	Establishment of a forest-loop merging a forest area and the school ground. The loop runs through the schoolyard and the forest and varies in the design as a consisting of a bench, a tribune, a broken climbing-ladders, balance-bars and a forest-portal. Along the loop different locations are found such as a forest-café, a pit-stop for mountain-bikers, a forest-arena, a forest jump, a playing field and a spider’s web.	• Movement policy
Grade 4-9		• New rules in recess
		• Longer recess periods
4	Creating a landscape for movement by establishing a learning/activity slope connecting the schoolyard and a forest area. The slope will contain learning locations with QR-codes supplemented with an App. The slope runs by several activity locations such as balance-bars, a climbing-net, swings in the trees, trampolines, a skating area, and an obstacle course.	• Longer recess periods
Grade 4-9		
5	Rebuilding a traditional flat asphalt covered schoolyard adding three different types of landscapes: 1. The mountain area consisting of several caves, a skate area and The”river delta” for water activities. 2. The forest area with trees, hammocks, and balance-bars. 3. The small-city area with small play houses.	• To be developed
Grade 4-6		
6	Building a simple 166 m^2^”super furniture” including platforms, canopy, stairs and a shed with basic equipment for playing, movement and teaching.	• To be developed
Grade 4-6		
7	Creating two main spaces for activity connected by running- and obstacle-trails: 1. A multi-court surrounded by activity gables, benches and learning trails. 2. Renovating the existing schoolyard adding a stage, a small hill with trampolines, markings on the asphalt surface, covering the existing walls with blackboards for drawing, teaching and ballgames.	• To be developed
Grade 4-9		

### Data collection and measurements

As described above, the study consists of different parts and each part has its own data collection method and measures, described in more detail below. Prior to the Activating Schoolyards Study a pilot study was conducted to test objective and subjective measurements of PA and classification of movement behavior patterns using accelerometers, global positioning system (GPS), questionnaires, class-diaries and interviews. Based on these findings small adjustments were made to improve the data collection procedure.

All parents of the students who participated in our study provided active informed consents, and all participants could withdraw from the study at any time. Data were collected in accordance with the Helsinki declaration. The study and its data-management procedures have been approved by the Danish Data Protection Agency (2013-41-1900 and 2014-41-2801).

#### Exploratory study

The aim of the exploratory study was to get an understanding of the students’ PA patterns and perceived barriers for PA during recess [26]. Non-systematic participant observations were conducted to gain insight in the students’ movement patterns, behavior and social interaction during recess [28] whereas interviews were carried out to gather in-depth data of the students perceived barriers for PA during recess [29, 30]. To facilitate the conversation and evoke memories the interviews were carried out in groups walking around in the schoolyard inspired by the go-along interview approach [31]. Data were collected during a one-day visit to the 17 cases selected for further development between April and June 2013. A total of 460 min of recess were observed. The observations were documented with field notes and photos [32]. A nominated teacher who knew the students was asked to identify three boys and three girls from fourth grade classes (10-11 years), representing different levels of PA. We recruited children representing different levels of PA to avoid stigmatizing of the least active children and to make generalizations of this group more reliable [33]. Seventeen go-along group interviews (one in each case) were conducted. In total 111 students (53 boys and 58 girls, mean age 10.4 years) participated in the go-along group interviews. The go-along group interviews lasted for approximately 60 min and were conducted during school hours.

#### Effect evaluation

The aim of the effect evaluation is to examine the effect of the tailored interventions in each of the cases on student’s PA and movement patterns during recess. Movement pattern is defined as PA intensity levels at specific geographic locations. The primary outcome is the difference in the objectively measured average activity level (in counts per minute, CPM) during recess in the schoolyard, before and after the intervention. The secondary outcomes are more exploratory examining intervention effects for the least active students, and exploring the change in behavior in specific areas of the schoolyard.

Baseline data were collected April to July 2014, and follow-up data will be collected in the same period (April to July) in 2016. A combination of accelerometers, GPS and geographic information system (GIS) was used to assess behavior changes in time and space in each of the seven cases. Objective PA was recorded as an activity-count every 15 s using the ActiGraph accelerometer model GT3X. The ActiGraph accelerometer has previously been recognized to provide acceptable validity and reliability for measuring children’s activity levels and energy expenditure [34, 35]. The students’ locations were measured every 15 s using QStarz BT-Q1000xt GPS trackers. The Qstarz GPS units have a median dynamic positional error of 2.9 m in real-world conditions, within various urban environments and during different modes of transport [36]. The schoolyards were mapped in detail using ArcGIS 10.2 and the total schoolyard area was calculated. During the week of measurements all participants completed an electronic survey, inquiring about self-reported PA, neighborhood and school experiences, and background characteristics.

The students were asked to wear the accelerometer and GPS in an adjustable elastic belt around their waist for seven consecutive days. The equipment was not worn overnight. Verbal and written instructions on wearing of the equipment were given to the students by the research team. To increase compliance short reminder text messages were sent out to the participants’ mobile phones twice a day. Two to three randomly selected participants in each class were asked to fill out a short timetable diary containing short questions about their school day and PA during class. Furthermore all schools provided detailed class time tables for the data collection period. At baseline the overall response rate was 52 % with 744 out of 1224 students in grade 4-8 participating. The response rate differed between school and class with a maximum rate of 82 %.

#### Process evaluation

The aims of the process evaluation were to facilitate the intervention development process and to identify barriers and facilitators in the implementation process. To help facilitate intervention development, the process evaluation was designed based on formative process evaluation principles [37, 38]. The process evaluation was carried out using an electronic survey to the 17 school principals from the cases selected for development in June 2013 and focus group interviews with the 7 final case teams were conducted in April 2014, and will be conducted in spring 2016. The survey included questions about rules and policies regarding recess, PA, outdoor teaching and activities outside school hours. Furthermore the schools were asked about their initial plans and expectations towards the process.

The focus group interviews included between 5 and 10 members of the final seven case teams and the interviews focused on the case teams’ experiences during the project development process and their expectations for the coming implementation process. The interviews took place at the intervention schools and lasted approximately 90 min. The second focus group interviews with the case teams in spring 2016 will provide insights to the organizational changes implemented in each of the cases, as well as the intervention implementation process.

#### Post-intervention user-evaluation

The aim of the in-depth post-intervention user-evaluation is to explore how, and by whom, the new elements in the schoolyard are used, within and outside of school hours. The study will also explore how students perceive the organizational and physical changes.

### Data analysis

#### Exploratory study

Upon completion of the exploratory study, field notes, interview transcripts and photos were ordered with the explicit purpose of identifying barriers influencing engagement in recess PA across the cases [39]. The data was coded and arranged under headings derived from the social-ecological model distinguishing natural, social, physical and organizational barriers [40].

#### Effect evaluation

The effect of the schoolyard interventions on PA will be assessed by calculating the difference in the objectively measured average activity level (in counts per minute, CPM) during recess in the schoolyard, before and after the intervention (Δ average CPM during recess) using multilevel modelling to account for the nested structure of the data (*i.e.* time points, students, class, school). The analyses will be adjusted for overall activity levels, age, gender and parents’ socio-economic status. Furthermore, analyses of changes in the proportion of time in sedentary, light and MVPA in the schoolyard will be calculated to exemplify change in activity levels post the interventions. To increase generalizability of the findings, the objectively measured average activity level at the intervention schools will be compared to objectively measure average activity levels of students during recess for approximately 40 other Danish schools. This data is or will be available from other studies conducted by our university department.

The analysis of the secondary outcomes will be more exploratory requiring new methods to clean and prepare useful variable based on combined accelerometer and GPS data. Examples of secondary outcomes are: areas generating high level of activity (CPM or MVPA) in the schoolyard, areas of the schoolyard most likely to encourage MVPA for different groups of students (boys/girls, high/low activity groups, age-groups), exploring routes of activity in the schoolyard.

#### Process evaluation

A descriptive analysis of data from the pre-intervention electronic survey was conducted to identify the organizational structure at the cases regarding recess and schoolyards policies, rules and practices prior to the intervention. The pre- and post-intervention focus group data will be analyzed as a whole using a thematic analysis strategy [29, 30]. Relevant themes across cases related to how the process was experienced by the case teams and school principals in the different phases will be extracted to identify barriers and facilitators.

#### Post-intervention user-evaluation

Upon completion of the post-intervention user evaluation, field notes, interview transcripts and photos will be analyzed using a thematic analysis strategy [29, 30]. Themes will be developed through a coding and re-coding process in order to identify commonalities and divergences in how the students perceive and use their schoolyard within and between cases [39].

## Discussion

The aim of this paper was to present the study design, case selection, intervention development and measurements of the Activating Schoolyards study.

Tailored interventions that consist of changes to the physical schoolyard environment as well as the organizational context will be implemented in seven cases. As there are many different factors that can influence the result of this type of interventions, evaluating the effect and generalizing findings to other situations is rather complex, and requires a multitude of methods. The participatory approach and case selection strategy make the study design novel in many ways, providing a series of benefits, but also some challenges that will be discussed in the next sections.

### Design

The design is quasi-experimental, using existing data for comparison. Over the last decade the majority of published recess intervention studies have used randomized control trials (RCT) or quasi-experimental designs [18–20, 41–43]. In contrast to the RCT design we purposefully selected the cases that were to receive an intervention, and will compare the results with data from other cases that were also not randomly selected. In principle, not using an RCT design reduces the internal validity of a study: the starting point for the intervention cases and the comparison cases is not necessarily the same and potential changes might not be (entirely) explained by the intervention. Comparing our results to objectively measured PA levels of students from up to 40 other Danish schools makes it possible to assess if changes occurring over time were the result of temporal trends or the intervention. As the comparison cases were not selected randomly, potential differences between intervention and comparison outcomes are at some risk for confounding or bias.

### Case selection

The main reason for purposefully selecting the intervention cases was to increase the external validity of our results. Our intention with the case selection strategy and intervention development was to optimize the conditions needed to create a highly motivating and involving process [23, 24]. With the use of this selection strategy, the intervention development process, and the substantial amount of funding allocated to the cases, we aimed at making our cases ‘critical cases’ [44]. Theoretically, this means that if we do not find an effect in the current cases, we will not find an effect using this process elsewhere [44]. However, even if the interventions are successful, we fully acknowledge that it will be difficult to implement this type of intervention on a large scale as this would require many resources. Nonetheless, we do think that evaluating the effect and exploring the change in behavior in relation to specific intervention elements in the schoolyard will lead to recommendations for schools undergoing schoolyard renovations at some point in the future. The division of students into groups with different activity levels gives us the opportunity to explore whether specific designs or constructions serve different groups better than other in the recess domain.

Reflecting upon our case selection strategy, we anticipated that the participating schools were highly motivated, and that the competition fostered many original ideas that had strong local support. Even though only seven cases were selected for realization, we expect that some of 106 schools that submitted a vision will, in some way, continue developing their schoolyard; just by entering the competition thoughts and processes were set in motion. Results from the evaluation of another project with a similar form of recruitment by competition point to this [45].

On a more critical note, we should mention that we as researchers only had an advisory role in the selection of the seven cases. The evaluation panel appointed by the Partnership behind the Activating Schoolyards Campaign made the final decision and even though clear selection criteria were set, personal preferences and interests other than selecting the most appropriate cases seen from a research point of view might have played a role in the case selection.

### Development of interventions

During the intervention development phase principles of Community-Based Participatory Research were used to develop tailored interventions. This approach has proven to be an effective and viable approach for addressing social and cultural health disparities in community-based interventions [27]. Based on our previous experiences with schoolyard interventions, we learned that tailoring an intervention to local needs and wishes, building on local engagement, was crucial to the success of the intervention [24]. A consequence of this participatory approach was the diversity in the intervention development process and the driving force behind the ideas. In line with a participatory bottom-up approach it was up to the schools to define their case teams, resulting in a variation in the representatives involved. In some cases one or two teachers were in charge, in other a school principal, in a few cases parents, and sometimes planners from a municipality. Also the extent of student involvement varied. All case teams received similar inputs from researchers to help develop their idea.

### Measurements

Using the mixed methods design including qualitative and quantitative methods is a strength, with the different types of data complementing each other [46]. Data collected in the first exploratory study were, apart from being used by the case teams to help develop the interventions, also used to develop the student questionnaire in the effect evaluation. The results from the effect evaluation will be put into perspective using the data from the post-intervention user-evaluation. A process evaluation with several data collection moments will shed light on factors influencing the implementation of the interventions. These results will help understand and explain the results of effect analysis.

A novel aspect of our study is using the combination of accelerometer, GPS and GIS in the effect evaluation to objectively determine where and how active the students are in the schoolyard, before and after the intervention. This type of data has to our knowledge, not been used before in longitudinal studies to evaluate schoolyard interventions [19, 20]. A number of cross-sectional studies have used similar measures to look at how schoolyard environments influence the activity patterns and intensity levels [47–50].

The combination of accelerometer and GPS is relatively invasive for participants, and this might be reflected in the relatively low participation rate (52 %). Compared to earlier studies using the systematic observation method SOPLAY [25, 51, 52], our method has the added advantage that each individual is identifiable, which means that it is possible to adjust the analyses for the overall PA level of the individual student as well as other personal characteristics [53]. Additionally, the combination of accelerometer, GPS and GIS facilitates comparing activity levels across different locations with different features, something that is not possible in studies using SOPLAY [25]. Another strength of mixing these methods is the opportunity to divide students into groups based on their objectively measured activity level and *e.g.* focus on the least active students. Finally, these methods have the potential to assess if the change in activity in the schoolyard is ‘relocated’ activity (*i.e.* the same activity, but in a different location), or a true increase in activity.

## Conclusion

Evaluating the effect and success of schoolyard intervention is complex and the Activating Schoolyards Study represents a new approach in the field of intervention research by its study design, case selection strategy, participatory development of interventions and the use of mixed methods. The study will provide unique insights in the role and importance of the participatory planning process, tailoring changes to local needs and wishes, as well as the success of specific schoolyard elements in attracting active users. These results can be used to guide school administrators in optimizing schoolyard renovation projects.

## Abbreviations

CPM: Counts per minute
GIS: Geographic information system
GPS: Global positioning system
MVPA: Moderate to vigorous physical activity
PA: Physical activity
RCT: Randomized control trials
